# Foreword: special issue on computational finance and economics

**DOI:** 10.1007/s12065-016-0148-z

**Published:** 2016-11-16

**Authors:** Anthony Brabazon, Michael Kampouridis

**Affiliations:** 1 Natural Computing Research and Applications Group, Smurfit School of Business, University College Dublin, Dublin, Ireland; 2 School of Computing, University of Kent, Medway, UK

Computational Finance covers a wide and still growing array of topics and methods within quantitative economics. The core focus has long been on efficient methods, models and algorithms for numerically demanding problems. The advent of new computational methods, together with the advances in available hardware, has pushed the boundaries of this field outwards. Not only can the complexity of investigated problems be increased, one can even approach problems that defy traditional analytical examination altogether. One major contributor of such methods is natural computing.

Natural computing draws its inspiration from phenomena and systems in nature, converts them into computer programs and algorithms which can then be applied in many real-world application areas, including computational finance. The rationale behind this approach comes from the parallels between economic systems and the real world: Financial environments are typically extremely complex with high levels of uncertainty, noise and dynamics. This is equally true for real environments, yet nature has produced many mechanisms to deal with these requirements. Successful strategies from the natural world could therefore serve as blueprints for solving problems in all sort of areas. Some of these methods are valuable supplements to existing techniques: optimization heuristics, e.g., can be used where traditional concepts failed or required unreasonable simplifications in the problem statements. Typical examples would be evolution and natural selection: individuals with superior features have a higher chance of outperforming their competitors and, eventually, passing these features on to their offspring. Analogously, evolutionary search and optimisation techniques have populations of candidate solutions to the (financial) problem at hand, and the better suitable one of them is, the better its chances that it replaces a weaker one and forms the basis for new (and further improved) ones. Akin to natural evolution, superior offspring will replace their not-so-good competitors and ancestors. Several papers in this special issue use evolutionary principles in this manner to develop populations that fit a pre-defined objective function as well as possible.

Agent-based modelling (ABM) has become a fruitful area of financial and economic research in recent years. ABM allows the simulation of markets which consist of heterogeneous agents, with differing risk attitudes and differing expectations to future outcomes, in contrast to traditional assumptions of investor homogeneity and rational expectations. ABM attempts to explain market behaviour, replicate documented features of real-world markets, and allows us to gain insight into the likely outcomes of different regulatory policy choices. ABM approaches form a good fit with natural computing methods as the latter can inform agent learning and adaptation capabilities.

The inspiration for this special issue arose in part to the success of EvoBAFIN 2016, which took place at the Evo* 2016 conference in Porto, Portugal (30 March–1 April 2016). EvoBAFIN has been running for ten years, and provides a forum for the presentation and dissemination of cutting-edge research in the application of natural computing methods to Business Analytics and Finance. A number of the papers in this special issue arise from substantially extended versions of papers originally presented at EvoBAFIN 2016. These have undergone the same rigorous, peer-reviewed, selection process as the other papers in this issue.

*Selecting and Estimating Interest Rate Models with Evolutionary Methods* by Maringer and Deininger demonstrates how a differential evolution (DE) algorithm can be used to create parsimonious models for the problem of modelling monthly interest rate series. The advantage of the authors’ proposed method is that the parameter selection and coefficient estimation is done simultaneously over each iteration of the algorithm. Experiments took place on two different types of datasets: artificial datasets, and empirical datasets, the latter consisting of interest rate series (both short-term and long-term) for the United States, the Euro area, and Japan. Results on 20 artificial datasets showed that the DE approach is well-suited for this type of problem. In addition, the experiments on the empirical series were able to identify parsimonious models that show dependencies and spill-overs across maturities and currencies.

*Anatomy of a Portfolio Optimizer under a Limited Budget Constraint* by Deplano et al. focuses on the problem of portfolio optimization. The difficulty of the particular problem investigated by the authors was having a limited budget constraint; this is a common constraint among investors who do not have large amounts of money available. A Multi-Layer Perceptron network is used to model the expected returns. Then, a simulation process uses the above neural networks as oracles to optimize the portfolio management strategy. A multi-objective evolutionary algorithm is utilized to find a Pareto-optimal solutions that take into account risk and reward. The proposed approach is tested on real world data from the New York, Milan and Paris stock exchanges and is able ot outperform a buy-and-hold strategy, as well as a random walk. Lastly, all models show significant profitability under the three datasets.

*On the Completity of the EI Farol Bar Game: A Sensitivity Analysis* by Chen and Gostoli, a sensitivity analysis is carried on an agent-based model of the use of public resources as manifested by the El Farol Bar problem. Three parameters related to size are examined: network size, neighborhood size, and memory size. The idea behind the use of the El Farol Bar problem is that economic efficiency and economic quality can lead to a good-society equilibrium. This can be achieved by the presence of agents with social preferences. In this paper, the authors look into the three size-related parameters mentioned above and investigate their effect on the agent-based model dynamics. Their findings lead to the conclusion that social preferences play an important role over all the sizes considered. In addition, the authors found that when the network size becomes large, the bar capacity parameter can also affect the model.

*Enhanced Multiobjective Population-Based Incremental Learning with Applications in Risk Treaty Optimization* by Cortes and Rau-Chaplin focuses on the problem of reinsurance contract optimization (RCO). Their goal is to obtain faster and higher quality solutions than the ones previously reported in the literature. To do this, they propose an enhanced multi-objective population-based incremental learning algorithm (E-MOPBIL), where they make several modifications to the traditional MOPBIL algorithm, such as removing slabs, creating a best sub-population, and not computing the worst buckets. Results showed that a better Pareto frontier was achieved. In addition, the proposed algorithm is up to 21.5% faster than MOPBIL when running sequentially on a single thread. In terms of the quality of the solutions, E-MOPBIL achieved higher quality solutions and better hypervolume.

*Characterizing Order Book Evolution Using Self-Organizing Maps* by Brabazon et al. investigates how electronic order books evolve over time. By utilizing self-organizing maps, the authors build clusters of common order book shapes, where shape is defined as movements in the bid-ask spread and changes in the depth of the order book at each price point. Experiments are conducted by using high-frequency data from four equities from the London Stock Exchange. Brabazon et al. uncovers several characteristics of the LSE order book evolution. Findings indicate that order books exhibit intraday seasonality, that clusters of order book shapes can be identified, and that the transition of order book shape is not random.

